# Gunshot Wound of Stomach and Kidney—Recovery

**Published:** 1872-09

**Authors:** J. W. Brooks

**Affiliations:** 857 Wabash Ave., Chicago


ARTICLE II.—*Gunshot Wound of Stomach and Kidney—Recovery.* Reported by J. W. BROOKS, M.D., 857 Wabash Ave., Chicago.

Dec. 31st, 1871. W. C., about 30 years of age, had taken an early breakfast, and with his brother had been engaged in completing a job as carpenters. Was in the possession of uniform good health. I think had served some three years in the Union army during the war of the rebellion. At 11 o'clock they returned to their boarding house to make up their bills. He very soon discovered a Derringer pistol, carrying a half-ounce ball, lying in a drawer near him, took it, blowed into two or three of the muzzles, withdrew it and pointed it towards the stomach. His brother remonstrated; he replied, no danger, and at that instant one barrel discharged (it being, I think, a six-barreled one), taking a downward, backward, and slightly lateral direction. He staggered, partly falling on a bed, rallied to his feet, ran down stairs and sank on a lounge. In five minutes the writer saw him. There was great nervous shock; the skin cool, clammy and moist, prostration great, constant nausea, pain in the stomach, restlessness, respiration feeble, and some thirst. Surface wound about two inches to the left of the centre of the sternum, having cut the size of the ball from the lower edge of the cartilages of the false ribs. Directed him removed to an upper room and laid recumbent, head low, a cloth wrung from hot water to be placed over the wound and to be kept constantly applied, small pieces of ice (as large as a small filbert) placed in his mouth. Imperative orders that no instrument touch the wound, bottles of warm water to the feet, absolute rest. 3 P. M., no reaction, very restless, much nausea, pulse not improved. Gave one dram of opiate solution (prepared from the dregs of opium that had been used in the preparation of officinal tinct. opii), with tartaric acid, with directions to repeat in two hours if necessary, which was twice done. 8 P. M., still sick at the stomach, no reaction. At 8.15 P. M., vomited between one and two pints of coagulated blood; at 9.10 P.M., passed about three pints bloody urine, resembling the blood that flows from the vein of an individual killed by lightning; a fourth dram was now given. At 10.20 P. M., reaction commenced feebly; from 11 P. M. to 3 A. M., slept quietly, and on awaking vomited a large quantity of blood, which was the last blood vomited; at 4 A. M., passed an ordinary urinal half full of fluid, mostly blood; from this time he urinated about once in six hours, blood always passing till the fifth day, when the urine was of a natural color and quality.

After the first sixteen hours he had no pain whatever, and slept well every night. At the expiration of forty-two hours he was allowed one teaspoonful of iced milk, in four hours two teaspoonfuls; this was gradually increased till on the fifth day he was allowed one-third of a tumbler of the iced milk every six hours. Not an untoward symptom occurred. The hot cloth wet, was kept applied up to Jan. 6th, 1872. No other medicament was used. The fourth day the bowels moved naturally. On Jan. 8th, he was removed to his home in the country. On or about May 1st, he returned to the city, and followed his occupation as a carpenter. On examination the 7th of May, I found the ball under the skin, about two and a half inches from the spinous processes of the vertebræ, and nearly outside the eleventh rib of the left side, having passed out of the abdominal cavity between the eleventh and twelfth ribs. The direction of the ball, the vomiting of blood, the passing of blood by the urethra, the character of the shock and prostration, point unmistakably to the cutting of the stomach and left kidney by the ball.

